# Short-Context Regulatory DNA Language Models with Motif-Discovery Regularization

**DOI:** 10.64898/2026.02.05.703637

**Published:** 2026-02-06

**Authors:** Aman Patel, Anshul Kundaje

**Affiliations:** 1 Department of Computer Science, School of Engineering, Stanford University; 2 Department of Genetics, School of Medicine, Stanford University

## Abstract

Self-supervised DNA language models (DNALMs) are typically trained at massive scale on whole genomes and long contexts. However, regulatory sequence features are sparse, heterogeneous, and dominated by poorly conserved flexible syntax of short motifs, which can be difficult to learn from genome-wide self-supervision. As a result, annotation agnostic, long-context DNALMs struggle to learn regulatory syntax and can underperform simpler baseline models on key regulatory tasks. We therefore introduce **ARSENAL**, a short-context masked DNA language model trained on a functionally enriched regulatory corpus and augmented with a novel regularizer than that encourages motif discovery. ARSENAL improves recovery of diverse transcription factor motifs *de novo* and prediction of regulatory variant effects in the zero-shot setting compared to other DNALMs. Incorporating ARSENAL embeddings also improves supervised chromatin accessibility prediction over strong ab-initio baselines across multiple cell types and yields improved regulatory variant scoring. Finally, ARSENAL serves as a practical generative prior, enabling targeted regulatory sequence design under downstream functional constraints.

All code can be found at https://github.com/kundajelab/regulatory_lm, and models and data can be found at https://sageb.io/4ZpEnk

## 1 Introduction

DNA language models (DNALMs) promise a unified foundation for genomic sequence understanding, variant interpretation, and functional sequence design. Motivated by the success of protein language models [29, 18, 16], recent work has scaled masked and autoregressive architectures to long contexts and trained them on one or more whole genomes. Representative examples include Nucleotide Transformer, HyenaDNA, DNABERT-2, Caduceus, GENA-LM, Evo, and Evo2 [25, 34, 12, 22, 2, 10, 7]. These models aim to learn general-purpose sequence representations by increasing model capacity and training corpus size, often with explicit support for long input contexts.

For DNALMs to be useful in practice, they must learn the sequence syntax of functional genomic elements rather than primarily capturing genome-wide background composition. The human genome spans ~ 3 × 10^9^ base pairs and contains diverse functional sequence classes embedded within a large fraction of neutrally evolving DNA. Broadly, functional sequence can be grouped into two major categories. First, protein-coding regions from ~ 20,000 genes (about 1.5% of the genome) encode transcripts and proteins. Second, non-coding cis-regulatory elements—estimated to cover roughly 5–20% of the genome—control when and where genes are expressed and thus shape cell-type-specific transcriptional programs [8]. Regulatory elements such as promoters and enhancers often act through short (6–20 bp) binding sites for transcription factors and other DNA-binding proteins, where motif affinity, spacing, and combinatorial arrangement collectively determine regulatory output.

Learning useful representations of regulatory DNA is particularly challenging for genome-wide self-supervision. In contrast to the dense and regular constraints in coding sequences, regulatory syntax is sparse, combinatorial, diverse, and strongly context dependent [24]. As a result, functional motif-scale patterns are rare relative to background composition and repetitive structure, and model learning can be dominated by non-functional sequence statistics. Consistent with this claim, multiple benchmarking studies report that whole-genome DNALMs often show limited performance on regulatory tasks such as variant effect prediction and regulatory activity inference, and may fail to capture nucleotide co-dependencies characteristic of functional motifs [24, 28, 19, 6, 30]. These gaps can persist even as models scale to very long contexts and large parameter counts, suggesting that scale alone does not guarantee learning of motif-scale regulatory grammar.

Several approaches have been proposed to inject additional biological structure into DNALMs. In particular, the GPN family of models (e.g., GPN-MSA and GPN-Star) leverages evolutionary information by incorporating multiple sequence alignments and/or upsampling conserved regions during training, improving variant effect prediction in many settings [6, 33]. However, MSA-based formulations may be less flexible when alignments are unavailable and can complicate sequence generation and synthetic design workflows. This motivates complementary strategies that bias learning toward regulatory function while preserving standard single-sequence modeling and generation.

Here we introduce ARSENAL (**A R**egulatory **SE**quence **N**ucleic **A**cid **L**earner), a short-context (350 bp) masked DNALM designed to learn regulatory motif syntax through two key principles: **targeted pretraining** on ENCODE candidate cis-regulatory elements (cCREs) [9, 20], and a **frequency-domain auxiliary objective** that encourages predicted likelihood profiles to emphasize motif-scale structure. Conceptually, this regularizer is inspired by Fourier-transform attribution priors that we previously introduced for supervised regulatory models, which penalize high-frequency components in sequence attributions during training to improve motif recovery and stability [31]; here we adapt the same motif-scale frequency bias to self-supervised masked reconstruction in a DNALM.

We evaluate ARSENAL across several biologically meaningful settings. We show that ARSENAL’s zero-shot likelihood landscape recovers diverse transcription factor motifs *de novo* and captures interpretable motif-scale dependencies; that it substantially improves zero-shot regulatory variant effect prediction relative to prior single-sequence DNALMs; that its embeddings improve supervised chromatin accessibility prediction and supervised variant scoring when used in sequence-to-function models; and that it serves as a generative prior for both realistic sampling and targeted regulatory sequence design under downstream constraints. Together, these results highlight that short-context, functionally targeted DNALMs with biologically informed priors can yield more useful representations of regulatory DNA than long-context genome-wide pretraining alone.

## 2 Results

### 2.1 The ARSENAL model: targeted pretraining with motif discovery regularization

We introduce *ARSENAL*, a masked DNA language model designed specifically to learn regulatory sequence syntax. ARSENAL is trained on the ENCODE consortium’s set of ~ 2.3 million candidate cis-regulatory elements (cCREs), a curated collection enriched for promoters, enhancers, and other regulatory loci [9, 20]. Training on this distribution substantially increases the density of functional signal relative to whole-genome pretraining, where regulatory syntax represents a small fraction of the training corpus.

**Figure 1:**
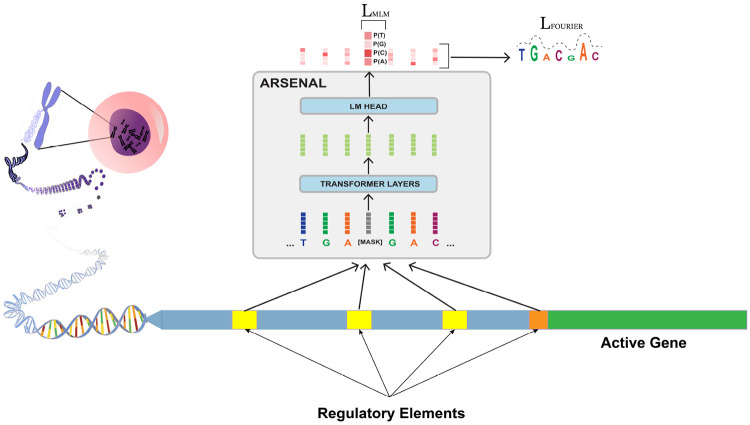
The ARSENAL model is trained on regulatory regions and augments the standard masked language modeling objective with a biologically informed Fourier transform-based loss [1]

In addition to standard masked language modeling (MLM), ARSENAL incorporates a **frequency-domain auxiliary loss** that biases masked reconstructions toward motif-like structure. Regulatory motifs are typically short (6–20 bp), whereas many non-informative genomic patterns are either very low-frequency (e.g., long repeats) or overly high-frequency (noise-like). The auxiliary loss serves as a biologically motivated regularizer that encourages ARSENAL’s per-base likelihood landscape to emphasize motif-scale variation (Fig. 1; Methods).

### 2.2 ARSENAL improves zero-shot motif discovery

An informative test of a regulatory DNALM is whether zero-shot likelihoods recover known motif-scale functional sequence features, i.e. whether the model assigns structured, high-confidence predictions within functional motifs while treating surrounding background sequence as comparatively uninformative. We evaluate this in two complementary ways: (i) case studies of locus-specific reconstructions and nucleotide dependency structure, and (ii) large-scale motif discovery benchmarks and aggregate motif statistics across many regulatory regions.

**Figure 2:**
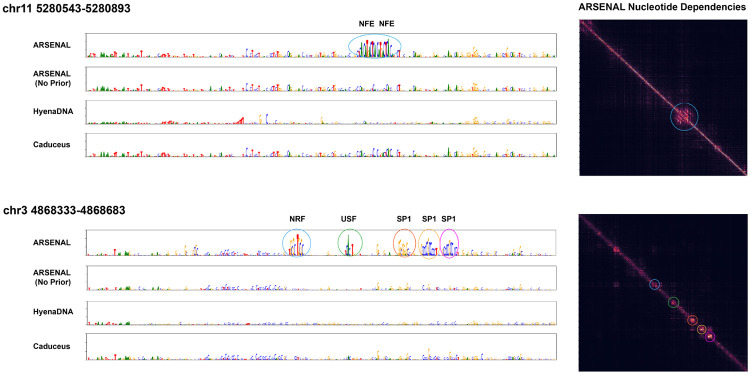
Language model reconstructions and ARSENAL nucleotide dependency plots of two example regions - an enhancers of the *β*-globin gene on chr11, and a cCRE with promoter-like signatures on chr3. Circled regions in the dependency plot are color-coded to correspond to motifs in the reconstruction tracks.

First, we visualize per-base likelihood reconstructions at representative loci and compare to those derived from other DNALMs. These likelihoods are computed by successively masking each position and predicting the probability of the true nucleotide given the remaining sequence context. Figure 2 shows predictions at two illustrative regulatory loci: an enhancer of the *β*-globin gene on chromosome 11, and a promoter-like cCRE on chromosome 3. In both cases, ARSENAL recovers a variety of known binding motifs at high confidence. For the *β*-globin enhancer, ARSENAL detects two key evolutionarily conserved MAFK/NFE motifs previously reported at this locus, while for the chromosome 3 promoter-like region it recovers multiple SP1, USF and NRF family motifs [21]. In contrast, an ARSENAL model trained without the Fourier regularizer (“ARSENAL (No Prior)”) fails to recover these motifs, indicating that the frequency-domain objective provides a substantial benefit beyond regulatory-region targeting alone. We further compare ARSENAL to HyenaDNA and Caduceus—the two single-nucleotide tokenized models evaluated in the DART-EVAL DNALM benchmark—and observe substantially weaker or less structured reconstructions in both cases.

To better characterize whether ARSENAL captures motif-level *dependencies* rather than only per-position confidence, we compute nucleotide dependency maps, which quantify the dependence of each prediction on nucleotides at other positions [30]. Ideally, positions within motifs should exhibit strong mutual dependencies and substantially weaker dependencies to flanking background. Indeed, ARSENAL dependency matrices show clear diagonal blocks aligned to the recovered motifs (Fig. 2), consistent with learning motif-scale features. We also often observe weaker off-diagonal blocks linking distinct motifs, suggesting that the model may additionally capture patterns of motif co-occurrence that shape context-specific regulatory activity.

**Figure 3:**
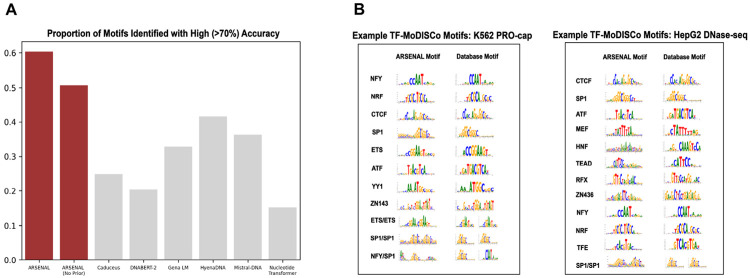
A) DART-EVAL motif identification task results for ARSENAL and several other DNA language models. B) Selected patterns from TF-MoDISCo results for K562 PRO-cap (left) and HEPG2 DNase-seq (right) peak sets. Modisco motifs are compared to canonical motifs from HOCOMOCO [32].

Second, we quantify the breadth and diversity of ARSENAL’s learned motif syntax using two large-scale analyses. We first apply the DART-EVAL motif identification benchmark[24], which measures how many known binding motifs a model assigns higher likelihood to than shuffled versions of the same motifs. ARSENAL substantially outperforms other DNALMs on this task (Fig. 3A), indicating that it preferentially recognizes a broad set of regulatory motifs in a zero-shot setting. Notably, ARSENAL (No Prior) still exceeds the performance of other DNALMs, consistent with the benefit of restricting pretraining to regulatory regions, while the full ARSENAL model achieves an additional gain from the Fourier motif discovery regularizer.

As a complementary analysis, we apply TF-MoDISco to likelihood-based contribution scores across hundreds of thousands of sequences to derive a consolidated set of motifs learned consistently across diverse regulatory contexts [27]. We analyze two regulatory collections: promoter regions associated with nascent transcription in K562 cells and DNase-seq chromatin accessibility peaks from HepG2 cells. In K562 promoters, TF-MoDISco discovers a wide range of canonical promoter-associated motifs, including NRF, NFY, ETS, and others (Fig. 3B), consistent with the model learning promoter syntax beyond simple base composition. In HepG2 DNase peaks, which span a broader range of enhancer and promoter elements, we recover a correspondingly broader motif repertoire, including important cell-type-associated families such as HNF, TEAD, and MEF2 alongside more general regulatory motifs.

Overall, these analyses demonstrate that ARSENAL learns a rich and biologically meaningful regulatory motif syntax in a purely self-supervised manner, both at individual loci and in aggregate across diverse regulatory elements. Moreover, ARSENAL’s likelihood reconstructions and nucleotide dependency maps provide an interpretable view of motif structure and interactions directly from the pretrained model, enabling mechanistic inspection of regulatory sequence regions without requiring task-specific supervision.

### 2.3 ARSENAL improves zero-shot variant effect prediction in regulatory QTL benchmarks

We next asked whether ARSENAL’s improved motif-level likelihood structure translates into better performance on variant effect prediction, a central application of regulatory sequence models. Beyond its practical importance for prioritizing non-coding variants, this task provides a stringent test of counterfactual generalization and calibration: the model must assign quantitatively meaningful probabilities at single-nucleotide resolution such that a single base change produces an appropriate shift in likelihood. Crucially, this sensitivity must emerge from sequence learning alone, as the model is not explicitly trained on variant annotations or allele-specific measurements.

We evaluate ARSENAL on the DART-EVAL zero-shot variant scoring benchmark, where single-nucleotide variant effects are computed using a likelihood-ratio score between the alternate and reference alleles [24]. Concretely, for masked language models, this corresponds to masking the variant position and comparing the model-assigned probability of the alternate base to that of the reference base given the surrounding context. This scoring is biologically intuitive for regulatory DNA because high-effect variants frequently act by creating, disrupting, or weakening transcription factor binding motifs, and motif perturbations should be reflected as sharp changes in position-specific likelihood under a model that has learned regulatory syntax.

**Figure 4:**
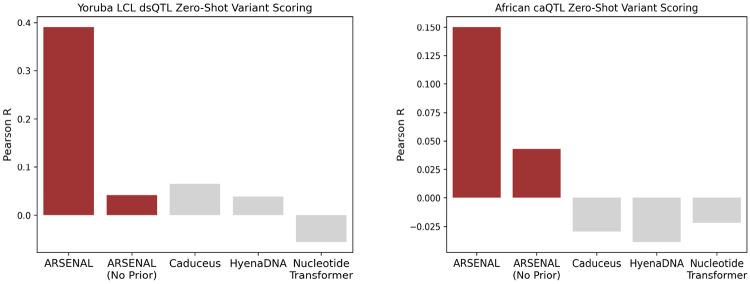
Comparison between ARSENAL and other DNALMs on zero-shot variant scoring for Yoruban LCL dsQL and African caQTL variant datasets. Scores for the DNALMs are provided by the DART-EVAL benchmark [24].

We consider two DART-EVAL regulatory QTL datasets in lymphoblastoid cell lines: dsQTLs, which quantify effects on chromatin accessibility measured using DNase-seq, and caQTLs, which quantify effects on accessibility measured using ATAC-seq [24]. These variants were derived from African population cohorts, providing a realistic distribution of common regulatory variation. Across both dsQTL and caQTL settings, ARSENAL achieves substantially higher correlation with experimentally measured effect sizes than other DNALMs evaluated in the same framework (Fig. 4). Notably, ARSENAL operates on only 350 bp of sequence context around each variant, whereas the original DART-EVAL evaluation uses substantially longer windows (2,114 bp), indicating that robust regulatory variant scoring does not necessarily require long-context modeling when motif-scale syntax is captured effectively.

Finally, ablation of the Fourier regularizer reveals that it is critical for variant sensitivity: ARSENAL (No Prior) shows a marked drop in performance and approaches the behavior of other DNALMs (Fig. 4). Together, these results support the conclusion that ARSENAL’s frequency-regularized likelihood landscape better reflects motif-scale functional structure, enabling accurate zero-shot prioritization of regulatory variants.

### 2.4 ARSENAL embeddings improve supervised sequence models of chromatin accessibility

While likelihood-based zero-shot scoring is useful, many practical applications require supervised sequence-to-function models that are trained to predict cell-type-specific regulatory and transcriptional readouts measured by diverse assays [4, 23, 3, 5, 17]. Such supervision is often essential because regulatory mechanisms are inherently context dependent: different cell types express distinct transcription factors and cofactors, leading to partially distinct motif lexicons and grammars, and the same sequence features can have different quantitative effects across assays (e.g., chromatin accessibility, TF binding, transcription initiation). We therefore asked whether ARSENAL’s learned representations provide a useful inductive bias for supervised regulatory prediction across cell types and assay modalities.

**Figure 5:**
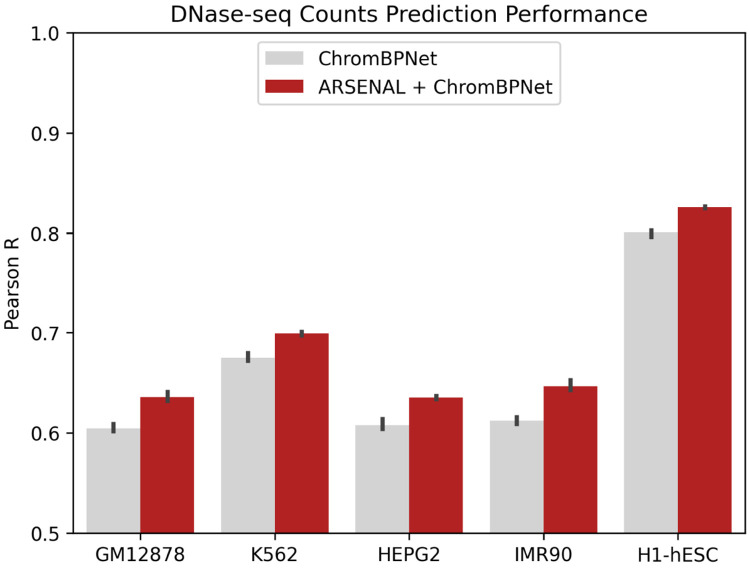
Comparison of counts prediction performance for ChromBPNet and a ChromBPNet-style model with ARSENAL embeddings as input. Models are trained on DNase-seq data from five different cell lines.

We adapted ChromBPNet, a state-of-the-art short-context sequence model of chromatin accessibility profiles [23], by replacing its one-hot input encoding with ARSENAL per-base embeddings while keeping the overall architecture and task unchanged (Methods). ChromBPNet predicts chromatin accessibility profiles from DNase-seq or ATAC-seq experiments over a 1,000 bp bins overlapping accessible peaks and background regions using 2,114 bp local sequence context. It achieves strong performance at predicting accessibility of held-out test sequences and downstream counterfactual variant effect prediction [23]. We trained embedding-based and one-hot baselines on DNase-seq data from five cell lines and evaluated performance on held-out test chromosomes at predicting total coverage across bins. Across all cell lines, ARSENAL embeddings improve prediction performance relative to the same ChromBPNet-like model trained directly on one-hot sequence (Fig. 5), indicating that ARSENAL embeddings capture regulatory features that transfer effectively to supervised chromatin accessibility prediction.

We next assessed whether these gains translate to the counterfactual setting of predicting regulatory QTL variant effects, estimated as the difference between model predictions for sequences containing the reference versus alternate allele. For models trained on the reference genome and DNase-seq data from the GM12878 lymphoblastoid cell line, ARSENAL-enhanced ChromBPNet improves variant scoring on the dsQTL and caQTL benchmarks used above (Table 1). This improvement is notable because performance on the supervised training objective does not necessarily correlate with performance on counterfactual variant effect prediction [24]. Together, these results suggest that ARSENAL’s self-supervised representations are sufficiently informative to improve supervised regulatory modeling and downstream variant interpretation pipelines.

### 2.5 ARSENAL can be used for targeted regulatory sequence generation and design

Finally, we evaluate ARSENAL as a generative model for regulatory DNA and as a practical prior for designing novel sequences with specified properties. Synthetic regulatory elements are increasingly important in applications ranging from therapeutic vector design and functional screening to systematic probing of cis-regulatory grammar[26, 11, 15, 13]. DNALMs provide a natural framework for this problem because they define an explicit sequence distribution that can be sampled or optimized to produce realistic regulatory DNA. However, the more demanding and practically relevant setting is *targeted* generation, where sequences must satisfy user-specified functional constraints rather than merely resemble the training distribution.

To test this capability, we combine ARSENAL with a user-defined objective function and perform iterative sequence optimization using a beam search procedure. Starting from an initial sequence, we repeatedly generate candidate edits by masking and resampling a specified fraction of positions for a fixed number of steps, then retain the highest-scoring candidates under the target objective. This design allows flexible control over the tradeoff between exploration and fidelity to the starting sequence, since masking rate and resampling depth effectively tune the similarity of optimized sequences to the original input (Methods). While the framework supports arbitrary objectives that return a scalar score, here we focus on regulatory design objectives defined by predicted chromatin accessibility levels from pretrained ChromBPNet oracle models in one or more cell types.

**Figure 6:**
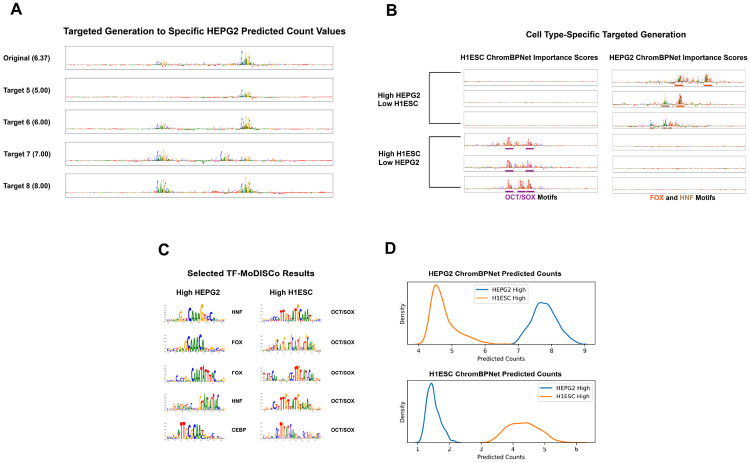
ARSENAL-aided targeted generation to produce sequences of specific properties. A) Example sequences optimized to produce specified predicted count values in the HEPG2 cell line. B) Example sequences optimized for differential predicted activity in the HEPG2 and H1-hESC cell lines with selected motifs color-coded. C) TF-MoDISCo results on ChromBPNet contribution scores for sequences optimized for differential predicted activity. D) ChromBPNet predicted count distributions for sequences optimized for differential predicted activity.

Using this approach, we first generate sequences optimized for graded levels of predicted accessibility in HepG2 (Fig. 6). The resulting sequences show a clear progression in predicted coverage, and interpretation of ChromBPNet contribution scores indicates that higher-activity designs preferentially introduce stronger or more numerous motif instances, including prominent CTCF binding motifs. We next optimize for cell-type specificity by generating sequences with differential predicted accessibility between HepG2 and H1-hESC cell-lines (Fig. 6). The two optimized sequence sets display clear separation in predicted accessibility across the two cell lines. Motif discovery using TF-MoDISco on contribution scores from the ChromBPNet models further reveals biologically relevant differences consistent with the design objective: HepG2-specific sequences are enriched for FOX, HNF, and C/EBP family motifs, whereas H1-hESC-specific sequences preferentially contain OCT4 and SOX2 motifs as well as combinatorial occurrences of both (Fig. 6C).

Together, these results demonstrate that ARSENAL can support efficient sampling and flexible targeted design of regulatory DNA when coupled with pretrained oracle models. Moreover, the combination of controllable optimization and motif-level interpretability enables inspection of the specific sequence features driving the designed functional profiles, making this a practical workflow for regulatory sequence engineering applications.

## 3 Discussion

ARSENAL demonstrates that a compact, short-context DNALM can learn meaningful regulatory sequence syntax when training is regularized with prior knowledge about regulatory biology. Two design choices are central: (i) pretraining on ENCODE cCREs concentrates learning on functional regulatory sequence and avoids dilution by genome-wide background, and (ii) a frequency-domain regularizer provides an effective inductive bias toward motif discovery, improving interpretability (motif recovery and dependency structure) and downstream utility (variant scoring and supervised transfer). Notably, these gains arise from sequence-only self-supervision, without requiring cell-type-specific functional labels.

A key methodological contribution of ARSENAL is importing Fourier-based attribution priors into DNA language modeling. Our prior work showed that penalizing high-frequency components in attribution maps during supervised training improves motif discovery and stability [31]; here we adapt the same motif-scale frequency intuition to self-supervised learning by constraining masked reconstruction behavior so that learned likelihoods emphasize motif-length features. This offers a simple way to softly bias DNALMs toward regulatory syntax without additional supervision, alignments, or explicit motif labels.

These results support a complementary perspective to long-context, whole-genome DNALMs: scaling context and corpus size alone does not guarantee learning of functional regulatory grammar when signal is sparse relative to background composition. For many downstream regulatory tasks, such as transfer learning and variant effect prediction, representations that prioritize local motif syntax can be more useful than representations dominated by repetitive or compositional features.

Several limitations remain. ARSENAL is cell-type agnostic and uses a 350 bp context window, which captures local motif grammar but not long-range dependencies such as enhancer–promoter coupling. More generally, DNALMs can learn non-functional sequence patterns (e.g. repeats) at high confidence, and sequence-only learning is constrained by motif frequency and context in the training distribution; supervised models trained on cell-type-specific functional tracks can more directly emphasize rare but active motifs. Our sequence generation results are also primarily evaluated *in silico* and warrant experimental validation.

Looking forward, this work suggests several directions. Additional priors could be incorporated as regularizers to encourage or suppress specific sequence features (e.g., repeat-aware penalties), and analogous priors may benefit other functional non-coding regimes such as splice regions or UTRs, though careful specification will be required to avoid suppressing relevant biology. ARSENAL could also be combined with conservation- or MSA-based objectives as in GPN-style models [6, 33] to integrate biochemical and evolutionary constraints. Finally, long-context DNALMs may benefit from initialization or modular augmentation with motif-regularized short-context representations, transferring robust local syntax while reserving long-context capacity for distal dependencies and higher-order genomic organization.

## 4 Methods

### 4.1 Pretraining Data

ARSENAL was pretrained on the ENCODE consortium’s official list of candidate *cis*-regulatory elements (cCREs) [9, 20]. This list comprises approximately 2.3 million high-confidence regulatory elements, spanning enhancers, promoters, and more ambiguous regions with regulatory signal. cCREs are identified and categorized according to sequencing experiments measuring chromatin accessibility (DNase-seq and ATAC-seq), histone mark occupancy (H3K27ac and H3K4me3 ChIP-seq), and transcription factor binding (CTCF and other TF CHIP-seq). Overall, the ENCODE cCREs represent the most extensive set of high-confidence regulatory elements curated for the human genome.

Data was split by chromosome. The validation set consisted of chromosomes 6 and 21, the test set consisted of chromosomes 5, 10, 14, 18, 20, and 22, and the rest of the genome comprised the training set.

### 4.2 A Biologically Informed Objective Function

We discovered that a biologically-informed objective function was necessary to effectively learn regulatory sequence features. We take inspiration from Tseng et al., who noted that the gradients of supervised regulatory sequence models are often confounded by noise and spurious high-frequency features, thus hampering the ability to fully learn the relevant functional syntax. A correction is made by applying a Fourier transform-based loss to the gradients during training, which specifically penalizes the presence of high-frequency components [31]. We adapt this objective to our model, except we apply it to the model’s output likelihoods rather than gradients, since these represent the main feature attributions for a language model. We also add a penalty for low-frequency components to de-incentivize learning uninformative long repeat sequences at high confidence.

More formally, we utilize the fact that most regulatory sequence motifs are between 6-20 bp in length, with features outside this range unlikely to be biologically meaningful. In Fourier space, broader features will concentrate energy at low frequencies, while shorter features will show greater density at higher frequencies [31].

As part of our masked language modeling objective, the model $f_{\theta }$ produces a vector of predicted probabilities from an input sequence x. We apply the discrete Fourier transform to this vector, obtain the the positive frequencies (excluding the DC component), and then L1 normalize the remaining values:

$$a={\left\|FFT(f_{\theta }(x))\right\|}_{1}$$

For original sequence length L, let $C_{H}=\frac{L}{6}$ and $C_{L}=\frac{L}{20}$ be our high and low-frequency cutoffs respectively. We then assign weights to each position i in a:

wi={11+(CL−i)si<CL1CL≤i≤CH11+(i−CH)si>CH}

where s is a smoothing factor hyperparameter.

Finally, our Fourier prior loss is given as:

$$L_{att}=1-\sum_{i=1}^{L∕2}w_{i}a_{i}$$

We combine this loss with the standard masked language modeling objective as follows:

$$L_{total}=L_{MLM}+\lambda L_{att}$$

Where λ is a loss weight hyperparameter.

### 4.3 Architecture and Pretraining

The ARSENAL model consists of three components:

- An embedder, which maps input tokens to input embeddings
- An encoder, which processes the sequence through several transformer layers
- A language modeling head, which maps these embeddings to token probabilities

The model’s *embedder* consists of a single embedding layer which maps the tokens A, T, G, C, N, and MASK to size-768 embeddings. The embedding for the N token is initialized as a vector of zeros and is not updated during training.

The model’s *encoder* consists of a transformer encoder architecture with 8 transformer blocks. Each block has an embedding size of 768, 8 heads, and a feed-forward dimension of 3072. RoPE is used to inject positional information in all layers.

The model’s *language modeling head* consists of a linear layer with output size 768 followed by GeLU, LayerNorm, and a final linear layer with output size 4.

The model is designed to process sequences of length 350, which is the maximum size of any cCRE. Any shorter cCRE was expanded to this length. We employed two data augmentation strategies in training. First, sequences were reverse-complemented with probability 0.5. Second, each sequence was shifted by a randomly chosen value between −50bp and 50bp.

We updated model weights based on predictions on 15% of training tokens. 80% of these were masked in the input sequence, 10% were randomly mutated, and 10% were left unchanged. We did not predict on any N’s in the input sequence.

Repeat regions represent a unique challenge for DNALMs, as language models will naturally learn them with high confidence, potentially obscuring informative regulatory features. We therefore did not update any weights based on predictions within contiguous repeat stretches of at least 30bp. These stretches were defined using lowercase letters in the reference genome. However, more work is required to determine the most optimal approach to handling these sequences.

Training took place for 150 epochs with a learning rate of $1e^{-4}$. The Fourier loss was assigned a weight of 0.002 with a smoothing factor of 5.

### 4.4 Zero-Shot Visualization and Motif Discovery

For our visualization or TF-MoDISco analyses, model likelihoods for a sequence were produced by masking each position in turn and utilizing the rest of the sequence as context to predict on the masked position. (For HyenaDNA, which is autoregressive, likelihoods were generated using a single forward pass without masking.) Likelihoods were then normalized according to the following formula, as per [14]:

$$p_{norm}=\frac{p}{{\bar{p}}_{nuc}}$$

Where ${\bar{p}}_{nuc}$ is the average predicted likelihood over the sequence for the nucleotide in question.

We performed TF-MoDISco on two datasets, both produced by the ENCODE consortium. Promoter regions were obtained by using peaks for PRO-cap, a sequencing method for transcription initiation, in the K562 cell line. We also obtained a set of DNase-seq chromatin accessibility peaks in the HEPG2 cell line. The relevant ENCODE IDs for these datasets are ENCSR261KBX and ENCSR149XIL respectively.

Peaks in these datasets are of variable length, and in all cases, we focused on the central 350bp of each region, where most motifs are located. In addition, to mitigate the presence of extraneous features in the final results, we omitted all sequences with contiguous repeat stretches of at least 50bp. Our analysis is still effective without this step, but as TF-MoDISco is dependent on feature enrichment, restricting to sequences without long repeats produces the most extensive results.

TF-MoDISco was run using the official repository. We utilized a window size of 350 and a maximum of 1,000,000 seqlets per metacluster.

### 4.5 Nucleotide Dependency Analysis

Our nucleotide dependency analysis followed the method introduced in [30]. As stated in that paper, the dependency $e_{i,j}$ between positions i and j in the sequence is given by:

$$e_{i,j}=\max_{k\in \{A,C,G,T\}}|\log_{2}\left(\frac{\text{odds}(n_{j}=k\;|\;n_{i}\neq k_{\text{ref}})}{\text{odds}(n_{j}=k\;|\;n_{i}=k_{\text{ref}})}\right)|$$

Importantly, no positions are masked when calculating nucleotide dependencies.

### 4.6 Zero-Shot Variant Effect Prediction

We utilized the zero-shot portion of the variant effect prediction task in the DART-EVAL benchmark. This task involves two datasets - a set of DNase-sensitivity QTLs from a Yoruban population, and a set of chromatin accessibility QTLs from an African population [24].

In both datasets, variants are defined according to their chromosome, position, reference allele $n_{ref}$, and alternate allele $n_{alt}$. Each variant has an experimentally derived effect size, which is obtained by calculating the difference in the relevant quantity between sequences with each allele.

We score each variant by first extracting the 350 bp sequence centered upon the variant position. We then mask that position and obtain predicted likelihoods using our model. Our score s is then given by a simple log-likelihood ratio:

$$s=log\frac{p_{\theta }(n_{alt})}{p_{\theta }(n_{ref})}$$

We then evaluate the quality of the variant scores by comparing to the observed effect sizes. Note that correlation metrics are only calculated on variants with significant observed effect, following the convention in the ChromBPNet paper (for which these datasets were initially curated) and in the DART-EVAL benchmark [23, 24].

### 4.7 Supervised Model Training

ChromBPNet is a dilated CNN model which is the state-of-the-art for chromatin accessibility prediction and variant scoring from local sequence context alone. The model accepts 2,114 bp input sequences, given as a four-channel one-hot matrix with one channel for each nucleotide. It outputs the total chromatin accessibility read counts over the central 1,000 bp along with the probability distribution of reads over that stretch.

For our model, we replace this one-hot representation with ARSENAL per-position sequence embeddings. The architecture and training scheme of our downstream ChromBPNet-style model are identical to the original except with two changes: the first convolutional layer must accept 768 input channels rather than 4, and we use a learning rate of $1e^{-4}$ rather than $1e^{-3}$.

ARSENAL embeddings for this task were derived by averaging the outputs of the last six layers of the model’s encoder. Additionally, since ARSENAL accepts an input of only 350 bp, the input sequence was split into chunks of size 350, with the central chunk comprising the center of the sequence. Embeddings for each chunk were obtained individually and then concatenated. As 2,114 is not evenly divisible by 350, embeddings for positions outside full chunks were calculated using the first/last 350 bp of the sequence.

We trained three sets of models on DNase-seq data from the GM12878, HEPG2, K562, IMR90, and H1-hESC cell lines. All training data is the same as from [24]. Note that for the fairest comparison, we trained regular ChromBPNet models using our own codebase as well. All models used predicted counts correlation as the metric for early stopping. The training, validation, and test splits comprised the same chromosomes used for pretraining.

### 4.8 Supervised Variant Effect Prediction

Variant scores for any supervised models in this study were calculated as initially described in the ChromBPNet study. We extract the 2,114 bp sequence centered at the position in question, and we create two versions: one with the reference allele at the central position, and one with the alternate allele at that position. Let us call these sequences $S_{ref}$ and $S_{alt}$ respectively.

For our supervised model $g_{\theta }$, we compute the model’s predicted accessibility read counts for each sequence, and we obtain our score s by calculating the log fold-change of the two values:

$$s=log_{2}\frac{g_{\theta }(S_{alt})}{g_{\theta }(S_{ref})}$$

The model’s scores are then evaluated in the same manner as described for zero-shot scoring. Models for the GM12878 cell line were used for these calculations.

### 4.9 Sequence Generation

We first describe our generation loop, which is the core of our objective-guided generation workflow. This algorithm requires the definition of three main parameters:

- $N_{i}$ - number of iterations of generation to perform
- $p_{m}$ - probability of masking a token during an iteration
- τ - temperature value to scale model logits

Given a pretrained ARSENAL model $f_{\theta }$, and a seed sequence S the algorithm proceeds as follows:

1. For each iteration in $N_{i}$:
  - Create a partially masked sequence $S_{m}$ by masking each token in S with probability $p_{m}$
  - Predict probabilities at masked positions with temperature-scaled logits: $P_{\theta }=\text{SOFTMAX}(f_{\theta }(S_{m})∕\tau )$
  - Produce a new S by sampling from $P_{\theta }$ at masked positions while keeping unmasked tokens the same

Given the above algorithm, we now describe our full workflow. This process requires an ARSENAL model $f_{\theta }$ and an objective function h, which could involve prediction using existing supervised models. The choice of h is flexible and task-dependent, and the parameters necessary for the function may vary.

In addition to the untargeted generation parameters $N_{i}$, $p_{m}$, and τ, we define two further parameters:

- $N_{g}$ - number of overall generation and evaluation steps to perform
- k - number of sequences to retain after each generation step

Our algorithm follows a beam search strategy and is as follows:

1. Create an array A of generated sequences, initially with a single seed sequence S
2. For each step in $N_{g}$:
  - For each sequence in A, perform untargeted generation (with parameters $N_{i}$, $p_{m}$, τ) to produce a new set of sequences. These are added to A
  - For each sequence in A, apply the objective function h
  - If A contains less than k sequences, continue to the next iteration. If not, only retain the top k sequences according to the objective function

For this study, our generations involved producing sequences with certain cell type-specific properties, as defined by predictions from the relevant ChromBPNet models. Specific objectives we utilized include:

- Optimizing for one cell type: Minimizing absolute error between predicted counts and a target value in the HEPG2 cell line
- Enforcing cell-type specificity: Minimizing absolute error targeting high predicted count values in the HEPG2 cell line and low values in the H1-hESC cell line, or vice versa

Note that due to the imbalance between ChromBPNet and ARSENAL input sizes, we operated on the central 350 bp of the ChromBPNet input sequence, where most motifs are located.

We can visualize and evaluate these generations using many of the same techniques described previously. The choice of the parameters $N_{i}$, $p_{m}$, and τ determines the diversity of the generated sequences. The number of iterations and the masking probability influence the level of deviation from the original sequence, and the temperature adjusts the confidence of the model’s predictions.

For our TF-MoDISCo analyses of the cell type-specific generations, we conducted 5 generation runs each way, with 100 sequences produced per run. We then calculated contribution scores and ran TF-MoDISCo using the established protocol as found in [23].

## Figures and Tables

**Table 1:** Variant scoring performance on Yoruban LCL dsQTL and African caQTL datasets for ChromBPNet and ARSENAL+ChromBPNet supervised models.

Model	Pearson R	Spearman R	AUROC
Yoruban LCL dsQTLs			
ARSENAL+ChromBPNet	**0.754 ±0.025**	**0.757 ±0.022**	**0.896 ±0.016**
ChromBPNet	0.729 ±0.011	0.755 ±0.020	0.888 ±0.012
African caQTLs			
ARSENAL+ChromBPNet	**0.654 ±0.003**	**0.678 ±0.002**	**0.764 ±0.009**
ChromBPNet	0.634 ±0.007	0.659 ±0.003	0.755 ±0.009
